# Study of the Role of CREB, BDNF, and VGF Neuropeptide in Long Term Antidepressant Activity of Crocin in the Rat Cerebellum

**Published:** 2017

**Authors:** Bibi Marjan Razavi, Mahdieh Sadeghi, Khalil Abnous, Faezeh Vahdati Hasani, Hossein Hosseinzadeh

**Affiliations:** a *Targeted Drug Delivery Research Center, Mashhad University of Medical Sciences, Mashhad, Iran.*; b *Department of Pharmacodynamy and Toxicology, School of Pharmacy, Mashhad University of Medical Sciences, Mashhad, Iran. *; c *Pharmaceutical Research Center, Pharmaceutical Technology Institute, Mashhad University of Medical Sciences, Mashhad, Iran.*; d *Department of Medicinal Chemistry, School of Pharmacy, Mashhad University of Medical Sciences, Mashhad, Iran.*

**Keywords:** BDNF, CREB, Cerebellum, Crocin, Depression, P-CREB, Saffron

## Abstract

Antidepressant activity of crocin, saffron main component, has been established before. Based on previous study, it is suggested that elevation in the levels of BDNF (brain-derived neurotrophic factor), CREB (cAMP response element binding) and VGF neuropeptide could be considered as one probable molecular mechanisms involved in antidepressant activity of long term crocin administration in the rat hippocampus. In this study we further investigated whether the antidepressant activity of crocin in long term administration was associated with alteration in these factors in the rat cerebellum.

Crocin (12.5, 25 and 50 mg/kg/day) and imipramine (10 mg/kg/day) were administered interaperitoneally for 21 days to rats. At the end of experiment, animals were sacrificed and cerebellums were dissected. BDNF, VGF, CREB, and phospho-CREB (P-CREB) protein and mRNA levels in the rat cerebellum were evaluated using Western blot and quantitative reverse transcription-polymerase chain reaction (qRT-PCR).

In the current study significant increases in mRNA and protein levels of VGF, CREB and (BDNF) in long term crocin treatment were not observed in the rat cerebellum. Although a slight increase was observed in protein level of P-CREB compared to normal saline, but it was not significant.

It is concluded that antidepressant activity of crocin might be partially mediated to CREB. Moreover, other factors rather than BDNF and VGF neuropeptides may alter following long term crocin treatment in the cerebellum. To understand the precise mechanism of crocin antidepressant effects in the cerebellum, longer duration of crocin treatment in further studies is recommended.

## Introduction

Depression is one of the most prevalent mental health problems. According to the document, 1 in 5 adults experience at least one episode of depression in their lifetime. Women are almost twice as likely as men to develop depression (1). Depression is a complex disorder which results partly via exposure to the chronic stress (2, 3). Over the past century, a large number of medications with different mechanisms of action such as modulation of monoamine levels, have been introduced to treat depression (4). As approximately 30% of the patients suffering from major depression are not completely treated with current antidepressant drugs, furthermore, these medications alter synaptic monoamine levels within hours (4), so it is suggested that the delay in clinical response may be attributed to neural adaptive mechanisms including alterations in synaptic plasticity, neurogenesis, and synaptogenesis (5, 6). Moreover, research has shown that cAMP response element binding (CREB) protein is up regulated and phosphorylated by antidepressant medications (7). Increase in the expression of brain-derived neurotrophic factor (BDNF) and VGF, has also been observed. The transcriptions of BDNF and VGF are CREB dependent (8, 9). Because of the side effects of antidepressant drugs including anxiety, sexual dysfunction and loss of appetite, inadequate response by these medications and also developing tolerance during treatment (10), there is a requirement for more efficient and less side effects treatments for depression. Recently, the use of herbal therapy has considerably increased in the world for the treatment of many diseases especially mood disorders (11).

*Crocus sativus* L. (*C. sativus*), commonly known as saffron, is a perennial stemless herb of the Iridaceae family (12). In addition to its usage as a spice and as a coloring and flavoring agent in foods preparation and cosmetics, it has been also extensively used in modern and traditional medicines (12-14). Main components of saffron which are responsible for its pharmacological effects including: crocins, the principle coloring agent (mono and diglycosyl esters of a polyene dicarboxylic acid, named crocetin) (13, 15), the glycoside picrocrocin which is a precursor of safranal and responsible for its bitter taste and safranal, a monoterpen aldehyde which is the deglycosylated form of picrocrocin and is responsible for the characteristic aroma of saffron (16). 

 According to several studies saffron and its constituents such as crocin exhibit different useful properties, including antioxidant (17), antitumor (18), antinociceptive and antiinflammatory (19, 21), antitussive (22), antidote (23, 24), antihyperlipidemic (25, 26), anticonvulsant (27), anti-anxiety (28) and hypotensive effects (29, 30). It could also reduce morphine withdrawal (31), morphine-induced conditioned place preference (32), and could improve memory and learning ability after chronic cerebral hypoperfusion (33).

Antidepressant effects of saffron have been reported both in traditional and modern medicines (12, 34). One study revealed that antidepressant effect of saffron was more effective than placebo in the treatment of major depression and it was as effective as imipramine in the treatment of mild to moderate depression (35). Recently Vahdati *et al*. (2014) showed that chronic crocin exposure to the rats exhibits antidepressant effects using forced swimming test (FST). Crocin also significantly increased the CREB, p-CREB, BDNF, and VGF protein expressions in the rat hippocampus (36). 

In addition to the hippocampus which plays a vital role in control of emotional and behavioral responses (37), there is increasing evidence that the cerebellum also exerts an important role in the control of emotion and cognitive processing besides its role in motor coordination (38, 39). So, the present study was investigated to evaluate the protein and transcript levels of CREB, BDNF, and VGF in the rat cerebellum in order to verify the role of these factors in the antidepressant effects of crocin in other regions of rat brain rather than hippocampus. 

## Experimental

*Chemicals*

Stigmas of *C. sativus* L. from Novin Saffron (collected from Ghaen, Khorasan province, Northeast of Iran) was obtained and analyzed in accordance to the ISO/TS 3632-2. Crocin was extracted and purified as previously reported (40). High Pure RNA Tissue (#12033674001, Roche,Germany) and EXPRESS One-Step SYBR® GreenER™SuperMix (#11780-200,Invitrogen, USA) kits were used for RNA extraction and qRT-PCR, respectively. To measure protein contents, Bio-Rad Protein Assay Kit (#500-0002, Bio-Rad, USA) was used. Imipramine hydrochloride obtained from Marham Daru, Iran. All chemicals used in this study were analytical grade and purchased from Sigma-Aldrich (Germany).

*Animals and treatments*

Adult male Wistar rats (weight 250–300 g) were provided by animal center (School of Pharmacy, Mashhad University of Medical Sciences). They were maintained on 12 h light/dark cycles and at a temperature of 23 ± 1 °C with free access to food and water. These conditions were maintained constant throughout the experiments. All animal experiments were carried out in accordance to Mashhad University of Medical Sciences, Ethical Committee Acts. Rats were randomly divided into five groups of 6 rats in each group, 1) Control (Normal saline); 2) Crocin 12.5 mg/kg/day; 3) Crocin 25 mg/kg/day; 4) Crocin 50 mg/kg/day and 5) Imipramine 10 mg/kg/day. The treatment was continued for 21 days. All materials were administered intraperitoneally (IP.) (36). 

*Western Blot analysis*

At the end of experiment, animals were sacrificed by decapitation. Cerebellum was dissected on ice immediately after decapitation. Then, brain specimens were rapidly frozen in liquid nitrogen and stored at -80 °C. Western blot analysis was carried out on protein extracts from the cerebellum for BDNF, VGF, CREB and p-CREB. Tissues were homogenized in the homogenization buffer containing Tris 50 mm pH 7.4, 2 mm EDTA, 10 mm NaF, 1 mm Na_3_VO_4_, 10 mm β- glycerol-phosphate, 0.2% W/V sodium deoxycholate, 1 mm phenylmethylsulfonyl fluoride (PMSF), and complete protease inhibitor cocktail (Sigma, P8340) using polytron homogenizer (POLYTRON^®^ PT 10-35, Kinematica, Switzerland) in ice and then were centrifuged at 4° C for 15 min at 10000× g. The total protein content in supernatants was determined using Bradford protein assay kit (Bio-Rad). Supernatants were stored at -80 °C until further use.

Protein Levels of BDNF, VGF, CREB and p-CREB were measured by immuno blotting analysis. Briefly, equal amounts of protein extracts (50µg) were loaded to SDS-PAGE gel. After electrophoresis, proteins were transferred to PVDF membrane. Membranes were blocked with 5% non-fat dry milk in Tris-buffered saline tween for 3 h Primary antibodies were rabbit monoclonal anti-serum against CREB (Cell Signaling, #, 9197), rabbit polyclonal anti-serum against VGF (Abcam, # ab74140), BDNF (Abcam, # ab46176), mouse monoclonal anti-serum against P-CREB (Ser133) (Cell Signaling, #9196), mouse and rabbit monoclonal anti-serums against β-actin, (Cell Signaling, # 3700 and # 4970). All antibodies were diluted 1:1000. Anti mouse and rabbit horseradish peroxidase labeled IgG (Cell Signaling, #7076 and #7074) were used as secondary antibody. Protein bands were visualized using an enhanced chemiluminescence (Pierce ECL western blotting substrate) and Alliance Gel-doc. (Alliance 4.7 Gel doc, UK). UV tec software (UK) was used to semi quantify protein bands. All protein bands were normalized against corresponding β-actin protein bands (37).

*Isolation of RNA and qRT PCR*

Total RNA of rat cerebellum was extracted using High Pure RNA Tissue Kit (Roche, Germany) according to the manufacturer’s instructions. The quantity and quality of the isolated RNA were assessed using NanoDrop 2000 UV-VIS spectrophotometer (Thermo Scientific, USA) and samples were stored at −80 °C until use. To measure transcript levels, step one thermal cycler (ABI) and Express one-step SYBR R Green ERTM kit (Invitrogen, cat # 11780-200) were used. Primers for CREB, BDNF, VGF and β actin were designed using Becon designer 7.8 (Biosoft, USA) and their specificity was confirmed by BLAST (NCBI). (http://www.ncbi.nlm.nih.gov/tools/primer-blast/). Primers were purchased from Metabion international AG, Germany (Table 1).

Melting curve analysis was performed to analyze quality of primers and products. The expression of target genes was normalized against β-actin. ΔΔCT method was used to measure fold increase of genes compare to control group.

*Statistical analysis*

Data are expressed as mean ± SEM. Statistical analysis was performed using one-way ANOVA followed by Tukey–Kramer post- test for multiple comparisons. The p-values less than 0.05 were considered to be statistically significant.

## Results

*Effect of crocin on protein and mRNA levels of BDNF in the rat cerebellum*

**Figure 1:**
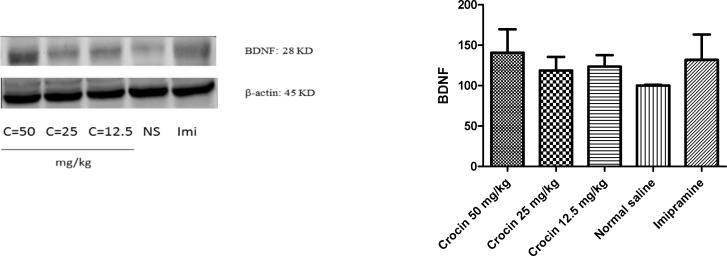
Effect of crocin on protein level of BDNF in the rat cerebellum tissue. (A) Representative western blots showing specific bands for BDNF and β-actin as an internal control. Equal amounts of protein sample (50µg) obtained from cerebellum homogenate were applied in each lane. These bands are representative of four separate experiments. (B) Densitometric data of protein analysis. Data are expressed as the mean ± SEM

**Figure 2:**
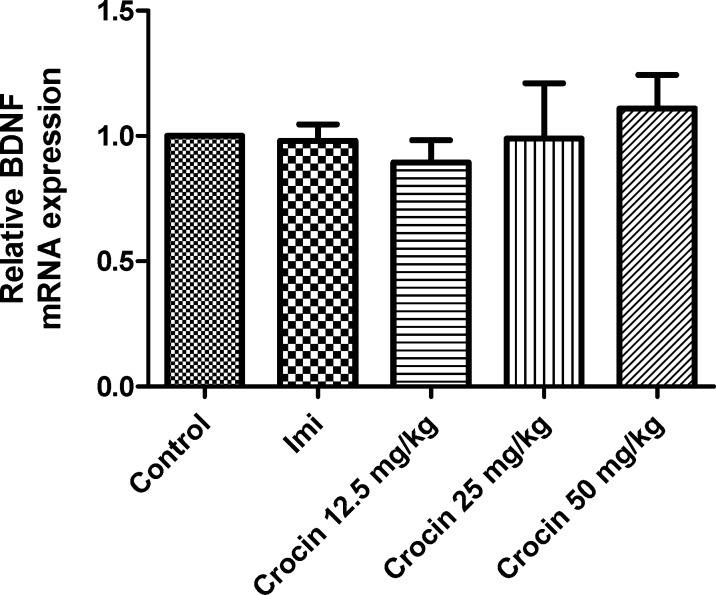
Effect of crocin on BDNF mRNA level in the rat cerebellum using real time PCR. The transcript level of each sample was normalized against β-actin transcript level. These reactions are representative of four separate experiments. Data are expressed as the mean ± SEM

Different doses of crocin and imipramine could not significantly increase protein (Figure 1) and mRNA (Figure2) levels of BDNF in comparison with the control group

*Effect of crocin on protein and mRNA levels of VGF in the rat cerebellum*

**Figure 3:**
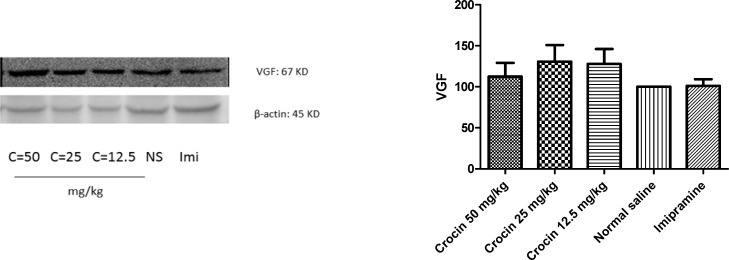
Effect of crocin on protein level of VGF in the rat cerebellum tissue. (A) Representative western blots showing specific bands for VGF and β-actin as an internal control. Equal amounts of protein sample (50 µg) obtained from cerebellum homogenate were applied in each lane. These bands are representative of four separate experiments. (B) Densitometric data of protein analysis. Data are expressed as the mean ± SEM

**Figure 4:**
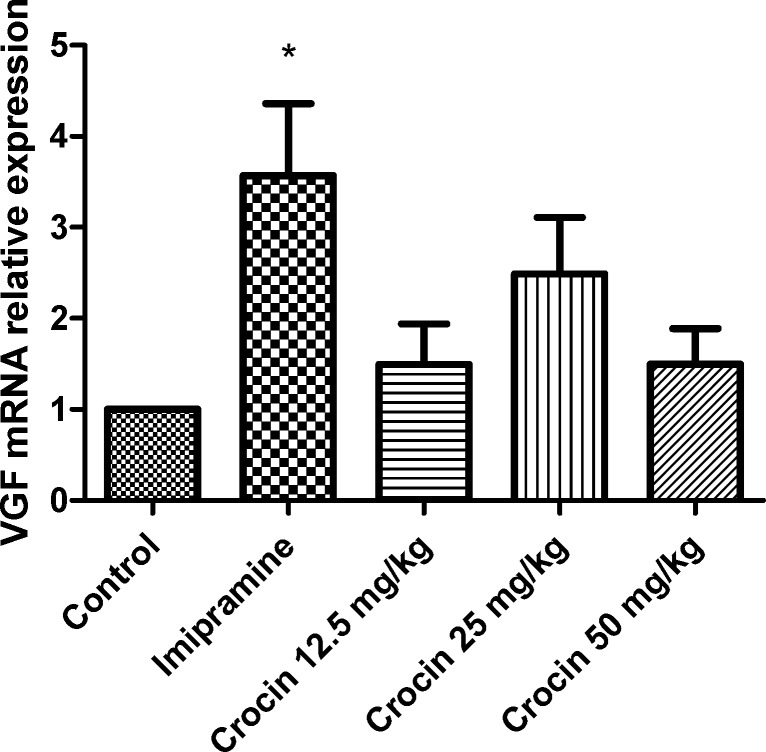
Effect of crocin on VGF mRNA level in the rat cerebellum using real time PCR. The transcript level of each sample was normalized against β-actin transcript level. These reactions are representative of four separate experiments. Data are expressed as the mean ± SEM. ^*^P < 0.05 vs control group

As shown in Figures 3 and 4 , different doses of crocin could not increase significantly the protein and mRNA levels of VGF in rat cerebellum. The results showed that imipramine significantly increased mRNA level of VGF in rat cerebellum in comparison with control group (*P*<0.05) (Figure 4).

*Effect of crocin on protein and mRNA levels of CREB in the rat cerebellum*

**Figure 5:**
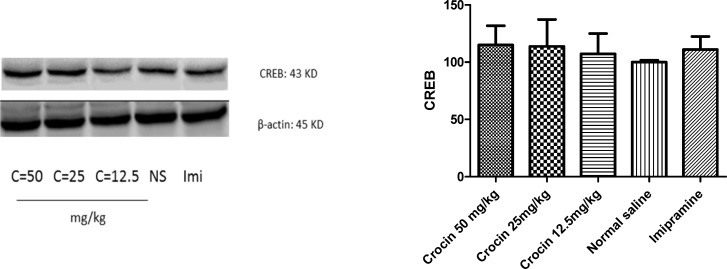
Effect of crocin on protein level of CREB in the rat cerebellum tissue. (A) Representative western blots showing specific bands for CREB and β-actin as an internal control. Equal amounts of protein sample (50 µg) obtained from cerebellum homogenate were applied in each lane. These bands are representative of four separate experiments. (B) Densitometric data of protein analysis. Data are expressed as the mean ± SEM

**Figure 6:**
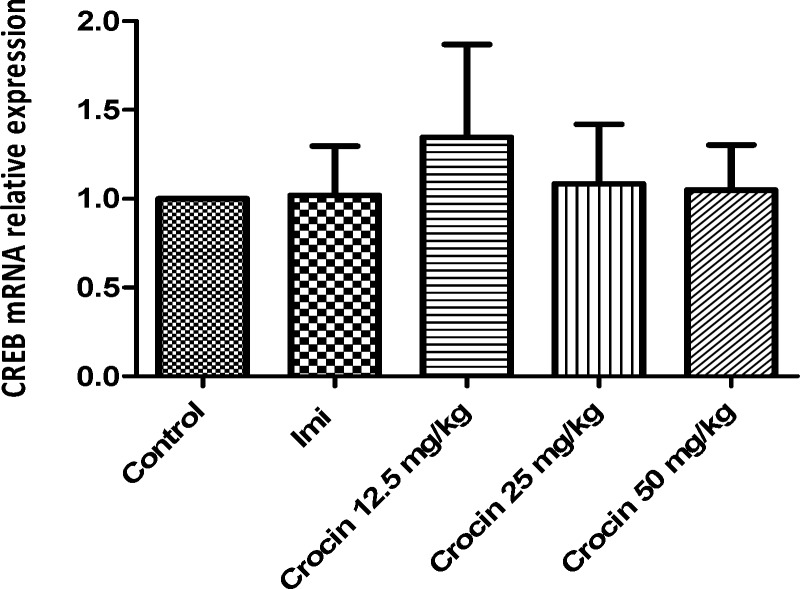
Effect of crocin on CREB mRNA level in the rat cerebellum using real time PCR. The transcript level of each sample was normalized against β-actin transcript level. These reactions are representative of four separate experiments. Data are expressed as the mean ± SEM. ^*^p < 0.05 vs control group

Different doses of crocin as well as imipramine could not alter significantly protein (Figure 5) and mRNA (Figure 6) levels of CREB in the rat cerebellum in comparison with the control 

*Effect of crocin on protein level of P-CREB in the rat cerebellum*

**Figure 7.**
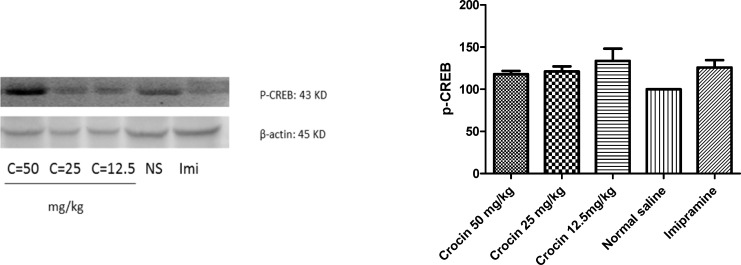
Effect of crocin on protein level of P-CREB in the rat cerebellum tissue. (A) Representative western blots showing specific bands for P-CREB and β-actin as an internal control. Equal amounts of protein sample (50 µg) obtained from cerebellum homogenate were applied in each lane. These bands are representative of four separate experiments. (B) Densitometric data of protein analysis. Data are expressed as the mean ± SEM

Although different doses of crocin and imipramine increased protein level of P-CREB in rat cerebellum, the effect was not statistically significant (Figure.7)

## Discussion

In this study, the role of BDNF, CREB and VGF neuropeptide in antidepressant effect of crocin on the rat cerebellum was investigated. Based on our previous study, we hypothesized that elevation in protein and mRNA levels of BDNF, CREB and VGF neuropeptide could be considered as one probable molecular mechanisms involved in antidepressant activity of long term crocin administration in the rat hippocampus. So, in this study we further investigated whether the antidepressant activity of crocin in long term administration was associated with alteration in these factors in the rat cerebellum. 

In the current study, significant increases in mRNA and protein levels of VGF, CREB, and BDNF in long term crocin treatment at different doses (12.5, 25 and 50 mg/kg) were not observed in the rat cerebellum. Although a slight increase was observed in protein level of P-CREB compared to normal saline, it was not statistically significant. 

**Table 1:** Sequences of different primers used for real-time PCR reactions.

**Gene**	**Sequence**		**Amplicon length (bp)**
**VGF**	**Forward** **Reverse**	**5`-GATGACGACGACGAAGAC-3`** **5`-CGATGATGCTGACCACAT-3`**	100
**β-actin**	**Forward** **Reverse**	**5`GGGAAATCGTGCGTGACATT-3`** **5`- GCGGCAGTGGCCATCTC-3`**	76
**CREB**	**Forward** **Reverse**	**5`-CCAAACTAGCAGTGGGCAGT-3`** **5`- GAATGGTAGTACCCGGCTGA-3`**	140
**BDNF**	**Forward** **Reverse**	**5`-TCTACGAGACCAAGTGTAATCC-3`** **5`- TATGAACCGCCAGCCAAT-3`**	152

Antidepressant activity of saffron and its active constituents, crocin and safranal have been established in previous studies in acute and chronic administrations (34, 41). Vahdati *et al.* (2014) showed that chronic administration of crocin at different doses increased immobility time in forced swimming test (FST) (36). FST is a common test used to study of antidepressant effects of different compounds in animal models (42). In this study, the effect of crocin was as the same as imipramine. Furthermore, a study by Hosseinzadeh *et al.* (2004) demonstrated that intraperitoneal administration of aqueous and ethanolic extracts of saffron stigmas and petals, as well as crocin and safranal, showed antidepressant effects in FST in mice. This study suggested that antidepressant effects of crocin and safranal could be related partly to the inhibition of dopamine, norepinephrine, and serotonin reuptakes, respectively (34). Akhondzadeh *et al*. (2004) evaluated the antidepressant effect of six weeks treatment by 30 mg/day saffron, three times a day, in patients with mild to moderate depression. The results of this clinical study revealed that antidepressant effect of saffron was more than placebo and the effect was as the same as imipramine (100 mg/day, TDS) (43). In animal studies, in order to evaluate the effect of saffron and its active components on motor activity, open field test was used and the results revealed that saffron and crocin have no effects on motor activity (34, 44). 

It is recognized that major depressive disorders (MDD) were associated with abnormalities in different brain areas. Moreover, alterations in expression of genes involved in neural adaptive mechanisms including those are responsible for synaptic plasticity, neurogenesis and synaptogenesis have implicated in the etiology and treatment of depression (11). Hippocampus, a brain area, possesses important roles in emotion, behaviour and also in response to the fear and anxiety (37). According to the documents, stress can induce death and atrophy of hippocampal neurons (45, 46). It is well known that hippocampal structural and functional changes are observed in antidepressant treatments, including synaptic plasticity, synaptogenesis, neurogenesis, and finally changes in mRNA and protein levels of CREB (cAMP response element-binding protein) and some neuropeptides such as BDNF (brain drived neurotrophic factor) and VGF (46-50). Although there are a lot of studies concerning the role of hippocampus in MDD, cerebellum abnormalities in depression has been reported in a few studies. Recent evidences reported that cerebellum possesses a critical role in control of emotion and cognitive processing besides its role in motor coordination (38). In depressed patients with cognitive impairment, regional cerebellar blood flow increases in the cerebellar vermis was identified (49). Furthermore, gray matter density reduction in the cerebellum has been observed (38). According to the study by Zeng *et al*. (2012), white matter microstructural abnormalities in depressed patients have been reported in some brain areas including the cerebellum (39). Moreover, functional connectivity between some cerebellum regions with different areas of cerebrum has been altered in geriatric depression (50). Based on our knowledge no study has evaluated alterations in neural adaptive mechanisms induced by some transcription factors and several neuropeptides including BDNF and VGF following antidepressant treatments in the cerebellum. This is the first report regarding the role of these factors in chronic antidepressant activity of crocin in the rat cerebellum. 

A large number of studies showed that neurotrophic factors, especially BDNF plays an important role in mental disorders. BDNF can modulate synaptic plasticity and it has a pivotal role in proliferation, development, and viability of both peripheral and central nervous systems (11, 51). Recent studies have shown that human BDNF level decreases in patients with schizophrenia, bipolar disorder, dementia and depression and antidepressants can increase its level (11, 52). There are strong evidences showing chronic administration of antidepressants increase BDNF mRNA level in the hippocampus of animal. According to studies, these changes are affected by some factors including duration of treatment and type of antidepressants (11, 53). It is proved that long-term treatment by serotonin reuptake inhibitors including citalopram, fluoxetine and paroxetine significantly increase BDNF mRNA level. In contrast, norepinephrine reuptake inhibitors have no effect on BDNF mRNA level both in acute and chronic exposures (54). Vahdati *et al.* (2014) showed that chronic crocin administration increased BDNF mRNA level in the rat hippocampus. According to their results, it is suggested that effect of crocin on BDNF mRNA level is similar to serotonergic antidepressant drugs (36). Ghasemi *et al*. (2014) also reported that chronic administration of saffron aqueous extract induced BDNF mRNA and protein levels in the rat hippocampus (41).

Unlike hippocampus, significant increases in BDNF protein and mRNA levels were not observed in the rat cerebellum following long term crocin administration. 

VGF, a polypeptide composed of 617 amino acids, has been found in different brain areas such as the olfactory system, cerebral cortex, hypothalamus, hippocampus, and spinal cord neurons (55). VGF neuropeptide regulates synaptic plasticity in hippocampus, in a manner very similar to that of BDNF through a tryosine receptor kinase B (TrKB) dependent mechanism. VGF mRNA level is highly affected by BDNF (56, 57). Increasing evidence suggests that VGF has a critical role in depression, so that it can induce antidepressant like properties in animal models (47). Significant elevations GF protein and mRNA levels in the rat hippocampus following chronic administration of crocin and aqueous extract of saffron, have been shown in previous studies (36, 41). Our results showed that crocin could not increase significantly VGF level in the rat cerebellum.

Critical role of CREB in neuronal survival and plasticity has been also identified (58). It is suggested that actions of neurotransmitters and neurotrophic factors on adult neurogenesis could be regulated by the cAMP-CREB cascade (59). The levels of CREB and phosphorylated CREB (P-CREB), an active form of CREB, reduced in post-mortem brains of depressed patients (60). Furthermore, the reduction in levels of CREB and P-CREB was observed in different animal models of stress in the hippocampus and frontal cortex (61). However, chronic administration of antidepressants increased CREB phosphorylation and activated it in rodents and human (48). Similar to BDNF, CREB is involved in regulation of VGF expression. CREB binds to VGF promoter and stimulates VGF expression (9). 

The results of previous studies on chronic administration of saffron aqueous extract and crocin in the rat hippocampus exhibited significant increases in CREB and P- CREB protein levels. These changes were associated with elevation in CREB mRNA level. Our results showed that a slight increase in protein level of P-CREB was observed in the cerebellum of rats exposed to crocin.

Regarding the critical role of CREB in MDD as a transcription factor which can affect on the several neurotrophic factors and different signaling pathways involved in the etiology of depression, so it can be concluded that other factors rather than BDNF and VGF neuropeptides may alter following long term crocin treatment in the cerebellum.

## Conclusion

Our results showed that a slight increase in protein level of P-CREB without any considerable effects on protein and mRNA levels of BDNF and VGF neuropeptides was observed in the cerebellum of crocin treated rats. Considering a slight increase in protein level of the activated form of CREB, it might be concluded that antidepressant activity of crocin is partially mediated to CREB. Moreover, other factors rather than BDNF and VGF neuropeptides may alter following long term crocin treatment in the cerebellum. To understand the precise mechanism of crocin antidepressant effects in the cerebellum, longer duration of crocin treatment in further studies is recommended. 
